# Morphological Signal Processing for Phenotype Recognition of Human Pluripotent Stem Cells Using Machine Learning Methods

**DOI:** 10.3390/biomedicines11113005

**Published:** 2023-11-09

**Authors:** Ekaterina Vedeneeva, Vitaly Gursky, Maria Samsonova, Irina Neganova

**Affiliations:** 1 Department of Physics and Mechanics & Mathematical Biology and Bioinformatics Laboratory, Peter the Great St. Petersburg Polytechnic University, 195251 Saint Petersburg, Russia; vedeneeva.ed@edu.spbstu.ru (E.V.); m.samsonova@spbstu.ru (M.S.); 2 Laboratory of Molecular Medicine, Institute of Cytology, 194064 Saint Petersburg, Russia; irina.neganova@incras.ru; 3 Theoretical Department, Ioffe Institute, 194021 Saint Petersburg, Russia

**Keywords:** human pluripotent stem cells, human embryonic stem cells, machine learning, best clone, morphological phenotype

## Abstract

Human pluripotent stem cells have the potential for unlimited proliferation and controlled differentiation into various somatic cells, making them a unique tool for regenerative and personalized medicine. Determining the best clone selection is a challenging problem in this field and requires new sensing instruments and methods able to automatically assess the state of a growing colony (‘phenotype’) and make decisions about its destiny. One possible solution for such label-free, non-invasive assessment is to make phase-contrast images and/or videos of growing stem cell colonies, process the morphological parameters (‘morphological portrait’, or signal), link this information to the colony phenotype, and initiate an automated protocol for the colony selection. As a step in implementing this strategy, we used machine learning methods to find an effective model for classifying the human pluripotent stem cell colonies of three lines according to their morphological phenotype (‘good’ or ‘bad’), using morphological parameters from the previously published data as predictors. We found that the model using cellular morphological parameters as predictors and artificial neural networks as the classification method produced the best average accuracy of phenotype prediction (67%). When morphological parameters of colonies were used as predictors, logistic regression was the most effective classification method (75% average accuracy). Combining the morphological parameters of cells and colonies resulted in the most effective model, with a 99% average accuracy of phenotype prediction. Random forest was the most efficient classification method for the combined data. We applied feature selection methods and showed that different morphological parameters were important for phenotype recognition via either cellular or colonial parameters. Our results indicate a necessity for retaining both cellular and colonial morphological information for predicting the phenotype and provide an optimal choice for the machine learning method. The classification models reported in this study could be used as a basis for developing and/or improving automated solutions to control the quality of human pluripotent stem cells for medical purposes.

## 1. Introduction

Assessment of the cellular morphology of biological samples has a long history, as it provides essential information on many underlying cellular processes and cellular states. In cell cultures, morphology is usually employed as a measure of cell classification; in clinical practice, morphological criteria are applied for diagnosis, prognosis, and treatment of human diseases. In recent years, quantification of cell morphology has seen great advances due to the development of new techniques and software that allow classification of cellular morphology from fluorescence or bright-field images at the single-cell level on both 2D and 3D cultures of cells on different substrates [1,2,3,4]. While fluorescent dyes may interfere with cellular functions, the live cell imaging under phase-contrast offers a great opportunity for label-free, non-invasive cell characterization and quantitative assessment of different parameters of cell morphology.

Nowadays, morphology-based high-content analysis of cellular phenotypes is increasingly recognized as a core methodology for the identification and analysis of cellular heterogeneity [1,5]. This is supported by the emergence of new software packages for high-dimensional image-based cell analysis with trained classifiers, such as CellProfiler Analyst, Enhanced Cell Classifier, and similar [5,6,7,8].

It has now been more than 15 years since machine learning (ML) and deep learning (DL) have granted us the computational power to understand questions in the field of cellular biology, drug development, medicine, etc. Without a doubt, research in the area of pluripotent stem cells, especially human embryonic stem cells (hESCs) and human induced pluripotent stem cells (hiPSCs), comprising human pluripotent stem cells (hPSCs), could benefit from the advances in ML and DL methods. These cells have the remarkable capability to differentiate to all the cell types of the human body, and these cells serve as a useful tool in regenerative medicine, disease modeling, drug testing, and the study of embryonic development. Two main types of hPSCs are very close in their morphology but have different origins. hESCs are derived from the inner cell mass of the preimplantation blastocysts, while hiPSCs originate through somatic cell reprogramming by overexpressing core pluripotency transcription factors [9,10]. Often, hPSCs are further differentiated into cell types that are useful for the researchers by subjecting them to a certain differentiation protocol. During this process, hPSCs undergo a global morphological transformation, in which the highly compact hPSC colonies give rise to more loosely organized cells with completely different morphological appearances and structures. Importantly, before the colonies from a single clone of the hiPSCs can be selected for further propagation followed by differentiation, these cells must be kept in culture in an undifferentiated state, without any signs of spontaneous differentiation.

Our group has a long-standing interest in developing an ML model for the best clone recognition based on the morphological parameters of the cells and colonies from hPSCs with different morphological phenotypes [11,12,13]. Although morphological changes can be quite evident to the trained human eye when colonies start to differentiate in an unwanted direction, this is inherently subjective and, thus, not applicable for the efficient translation of the laboratory methods to automated cell production for clinical purposes. Traditional manual cell culture is variable and labor-intensive, posing challenges for high-throughput applications. Moreover, the selection quality depends on the professional knowledge and practical experience of an expert, which limits the application of the manual feature selection method for cell culture assessment. In this regard, it is important to emphasize that the effective definition of morphological parameters and the evaluation of the extent of morphological heterogeneity within hPSC populations remain challenging.

Due to the huge expansion and wide use of hiPSCs in recent years [14], there is a need for new technologies to not only standardize the evaluation of iPSCs to allow the objective comparison of results across different groups, but also to ensure safe translation of these cells towards clinical use. Nowadays, regenerative medicine products are at the forefront of scientific research and clinical translation, but their reproducibility and large-scale production are compromised by poor automation, monitoring, and standardization issues, resulting in an increased batch-to-batch cell culture variability. To overcome these limitations, new technologies have been proposed at both software and hardware levels. Software solutions include algorithms and artificial intelligence models and are combined with imaging software and ML techniques, whereas hardware is presented by automated liquid handling devices, automated cell expansion bioreactor systems, automated colony-forming units, counting and characterization units, and scalable cell culture plates.

**Figure 1:**
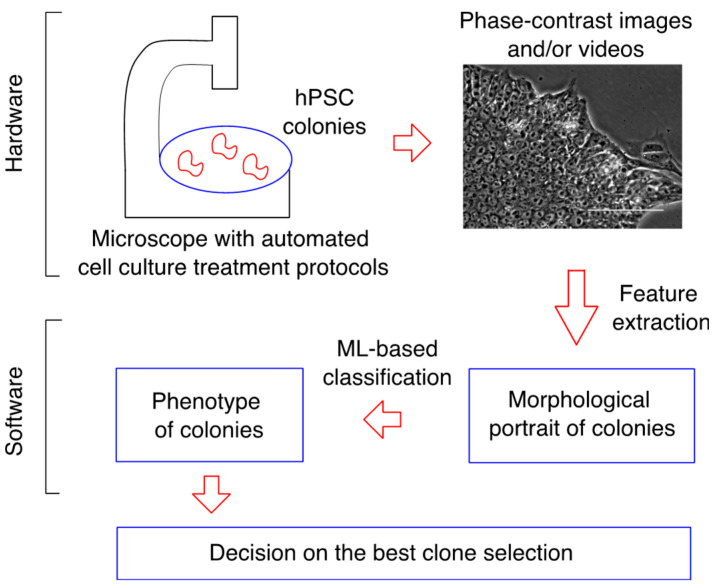
Schematic representation of a device designed for the automated best clone selection. Red arrows indicate the direction of information processing within the device.

As an example of such technologies, we illustrate in Figure 1 a conceptual schema for a device designed to select the best clone by controlling the quality of the hPSCs. The experimental part (‘hardware’) contains a microscope making phase-contrast images or videos of growing hPSC colonies on a substrate. The software consists of two parts. The first part extracts informative morphological features of cells and colonies from the images or videos, thus providing the morphological portrait of a colony. The second software part processes this morphological signal by applying to it pretrained ML-based models, yielding the assessment of the colony phenotype. Finally, using this information, a decision is made about whether the colony should be kept in culture for further propagation or terminated. Our work contributes to an important step in this schema related to the development of the phenotype prediction models.

There have been many efforts to utilize ML and DL methods to predict a hPSC phenotype and, thus, to provide the selection of the best clone [13]. These studies can roughly be split into two major classes. The first one comprises the phenotype classification models based on two-stage processing of the imaging data, in which biologically interpretable morphological features are first extracted from the hPSC images and then classification methods are applied with the extracted features as predictors [11,15,16,17,18]. Studies from the second class apply DL methods (e.g., convolutional neural networks) directly to the colony images to infer the phenotype, or some less biologically interpretable features are automatically extracted using image processing methods followed by ML-based classification with these features as predictors [12,19,20,21,22,23,24]. Despite the fact that the second approach often provides higher phenotype prediction accuracy, the first approach has an advantage in a clear biological interpretation of valuable morphological parameters found during this study, thus providing insights for possible new biological experiments. Various authors used different ML methods for phenotype prediction models, and their performance varies. Therefore, a search for a method that is optimal for a given datum is an important task.

The aim of our study was to identify the best classification method for predicting the hPSC colony phenotype based on morphological parameters of cells and colonies from three hPSC lines, given in the previously published data set [11,25]. As a further step, we proposed a model based on the combination of cellular and colonial parameters and showed that this model provided the best performance. Finally, we analyzed the importance of the morphological parameters in the resulting classification models.

## 2. Materials and Methods

### 2.1. Data

For model training, we used previously published data containing values of morphological parameters of cells and colonies extracted from phase-contrast images of three cell lines: human embryonic stem cell line H9 (WiCell, Madison, WI, USA), hiPSC line AD3, and patient-specific hiPSC line HPCASRi002-A (CaSR) [11,25]. The morphological parameters were as follows: area of the cell or colony (‘Area’), length of the cell or colony boundary (‘Perimeter’), length of the minor axis of the ellipse fitted to the cell or colony in the image (‘Minor axis’), largest distance between two points on the cell or colony boundary (‘Feret’s diameter D’), smallest distance between two points on the cell or colony boundary (‘Minimal Feret’s diameter D’), area divided by squared perimeter and multiplied by 4π (‘Shape factor’, a measure of circularity and compactness), and total area of the free intercellular space in the colony (‘Area of intercellular space’, a measure of compact cell packing within a colony). These parameters could be considered as standard shape descriptors in 2D image analysis using ImageJ software, version 1.54g [26]. The parameter values were obtained for 53 colonies and 1602 cells of hESC line H9, 49 colonies and 1569 cells of control hiPSC line AD3, and 48 colonies and 1315 cells of patient-specific hiPSC line CaSR [11].

All colonies and cells in the data set contained binary phenotype score obtained via an expert analysis, as previously described [11]. The binary phenotype score can take one of two values, ‘good’ or ‘bad’, representing the pluripotency status of the colony. Colonies with the good phenotype demonstrate a high potential for proliferation, while colonies with the bad phenotype show signs of spontaneous differentiation.

### 2.2. Classification Models

We used the data set for training classification models to predict phenotype based on the morphological parameters as predictors. The following classification methods were tested: naïve Bayes classifier, *k*-nearest neighbors, logistic regression, random forest, support vector machines, and artificial neural networks. We analyzed the classification problem for the cellular and colonial data separately. In addition, we combined the cellular and colonial morphological parameters and phenotypic information into a separate data set, which we call a combined data set, and trained classification models on these data. The predictors in the models for the combined data included morphological parameters of a cell and morphological parameters of the colony containing that cell, and the phenotype of the colony containing that cell was used as the target for classification. All models were implemented using Python 3.8 (sklearn and keras libraries) and trained using the nested cross-validation, with 5 folds in both inner and outer cross-validation loops [27]. In each fold of the outer loop, data were split into training and test sets. Then, the selection of hyperparameters occurred in the inner loop using cross-validation on the training set from the outer loop. The classification accuracy of the best model from the inner loop was estimated on the test set in the fold of the outer loop, which we called the nested cross-validation accuracy. The mean nested cross-validation accuracy ± s.d. was recorded for test sets of all outer loop folds. The neural network configuration was tuned manually, and the hyperparameters of all other methods were selected using the grid search method [28].

In addition to the accuracy, we recorded the Area Under Curve (AUC) for the Receiver Operating Characteristic (ROC-curve) as another effective measure of binary classification for the best classification model of each classification method. This measure represents the area under the curve on a plane with the true positive rate on the ordinate axis and false positive rate on the abscissa axis, with AUC = 1 representing perfect classification and AUC = 0.5 representing random classification.

### 2.3. Feature Selection

We analyzed the importance of each feature as a predictor in the best classification models by applying the SHAP method (SHapley Additive exPlanations) [29]. The SHAP value for each feature represents the contribution of that feature to the prediction value of the selected model. SHAP are theoretically well justified and unify several previously suggested methods. This analysis was implemented using shap library in Python 3.8.

### 2.4. Statistical Methods

We compared the average nested accuracies of the classification models for different classification methods using *t*-test (ttest_rel function, stats module, scipy library in Python 3.8).

## 3. Results

### 3.1. Classification Models for Cellular and Colonial Data

To find out how various classification methods perform on the morphological data for hPSCs and colonies, we estimated the cross-validation accuracy of phenotype prediction for each method using the data containing all cell lines pooled together (Table 1).

The results for cellular data showed a similar performance across various models, but the neural networks outperformed all the methods except the support vector machines (*p* < 0.05), with an average accuracy of 67%. Considering that the AUC measure was also the highest for this method, we can conclude that the neural networks method was the best model for predicting phenotype based on the morphological parameters of cells.

In the case of colonial data, the difference between methods was more pronounced, but also showed higher method-specific variation. Based on the average accuracy and the AUC value, logistic regression appeared to be the best classification method (75% accuracy) for predicting phenotype from the morphological parameters of colonies. Overall, the performance measures shown in Table 1 could be estimated as rather moderate, implying that either combining different cell-line-specific data in one data set or consideration of only cellular or colonial morphological parameters separately was possibly not an optimal strategy.

As the unification of various cell lines into one data set might create irrelevant variability, impeding the classification by phenotype, we tested whether the performance could be improved by considering each cell line separately. For this purpose, we trained classification models on the line-specific cellular and colonial data using the best classification methods from the analysis of the unified data (Table 2). These models incorporated the same morphological parameters as the models from Table 1 but were trained and analyzed on data of each cell line separately. The results showed an average performance for H9 that was either comparable to or higher than that for the unified data, but the models predicted the phenotype with less accuracy for other cell lines. Therefore, constraining the classification problem to the line-specific data did not improve the performance. These results showed that combining morphological data from different cell lines was justified, as it provided a larger data set without significantly degrading classification performance.

### 3.2. Classification Models for Combined Cellular and Colonial Data

Another way to improve the performance of the classification models in Table 1 was based on a biological hypothesis that colony phenotype could not be determined solely based on cellular or colonial morphological parameters. Cells differentiate irregularly within a colony, sometimes demonstrating a reverse behavior, so the phenotypic status is rather a collective property, also expressed in a change in the colony morphology. Under spontaneous differentiation, the morphological perturbations of both single cells and a colony as a whole should be considered as necessary elements of the true morphological portrait associated with the pluripotency potential.

**Figure 2:**
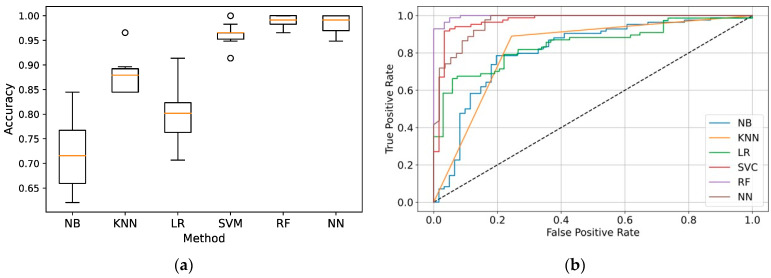
Performance of classification models trained on the combined cellular and colonial data: (**a**) Box plots for the nested cross-validation accuracy. Orange lines show the median accuracy values, boxes represent the interval between the lower and upper quartiles, whiskers mark the minimum and maximum accuracy values, and circles are outliers; (**b**) ROC-curves. The dashed line represents a random classifier, which assigns phenotypes randomly. NB: naïve Bayes; KNN: *k*-nearest neighbors; LR: logistic regression; SVM: support vector machines; RF: random forest; NN: neural networks.

Therefore, we tested the same classification methods but for the combined data set, in which predictors included morphological parameters of cells complemented with the parameters of the colony containing these cells. The results demonstrated a significant increase in performance for all methods (Table 3, Figure 2). The discrepancy between methods was also higher, with a more than 25% difference in the mean accuracy between the best and worst methods. Random forest and artificial neural networks showed the highest performance, which was clearly distinguishable from other methods (*p* < 0.05).

### 3.3. Importance of Morphological Parameters in Classification Models

**Figure 3:**
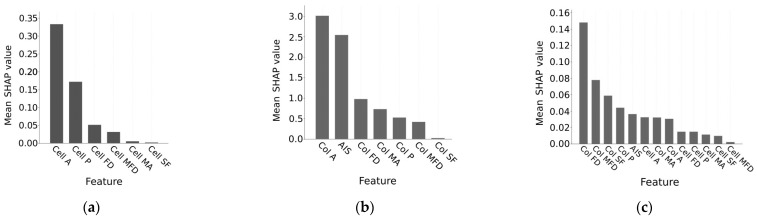
Mean SHAP values representing the importance of the morphological features in the best classification models based on (**a**) cell data, (**b**) colony data, and (**c**) combined data. Names of cellular parameters start with ‘Cell’, and names of colonial ones start with ‘Col’. A: Area; P: Perimeter; MA: Minor axis; FD: Feret’s diameter D; MFD: Minimal Feret’s diameter D; SF: Shape factor; AIS: Area of intercellular space (only for colonies).

The classification models that we obtained can be used to understand which morphological characteristics of individual hPSCs and colonies are the most informative in representing the morphological signal as a manifestation of phenotype. We used the SHAP method to find the features that were the most important in all types of classification models. In the best cell-data-based model, two parameters clearly segregated from the others: Area and Perimeter (Figure 3a). In the best model based on the colonial parameters, Area and Area of intercellular space were the most important for phenotype prediction (Figure 3b). For the combined data, the analysis showed that the colonial parameters appeared to be more important in the best classification model than the cellular parameters (Figure 3c). The colonial Feret’s D showed the highest impact on the classification, while other parameters exhibited a rather shallow distribution of their importance score. In other words, colonial Feret’s D can be considered as the most influential parameter in the classification models on the combined data, but other parameters also contributed. The cellular area had the highest importance score among the cellular parameters in this case (Figure 3c).

## 4. Discussion

For numerous cell types of the human body, their morphological appearance is mainly known and often described in terms of the cell size, cell form, its granularity, cytoskeletal architecture, etc. In many ways, these features of the cell morphology result from the spatiotemporally regulated activity of signaling proteins. However, the components of these signaling networks and the precise role they play in regulating the cell shape and other morphological parameters remain largely unclear. How and which signaling cascades govern the transition of the small pluripotent stem cell into a specialized and often much bigger cell type is still in question. In this regard, morphological profiling and identification of genes and clusters of genes which are important for maintaining hPSC morphological identity, as well as genes involved in the conversion of these cells into differentiated specialized cells, is undoubtedly important for both understanding hPSC biology and for the development of efficient protocols for directed differentiation. In this way, morphological data from our assay, together with novel computer-based assessment, can provide a further step toward discovering new biological connections that determine a hPSC’s identity.

Our results showed that cellular and colonial data required different classification methods, emphasizing the inherent data dependency of ML approaches. The classification quality of artificial neural networks for the cellular data was comparable with a value previously obtained by us for the same data using a similar method [11]. However, in contrast to that study, we found that logistic regression was more efficient when the morphology of hPSC colonies was considered. For the combined cellular and colonial data, random forest appeared as a promising approach, and the resulting classification model showed the best performance. This indicates that the true morphological portrait associated directly with the hPSC pluripotency should be assembled from both the morphological parameters of pluripotent cells forming the colony and the parameters of the colony as a whole.

We demonstrated that parameters such as Area and Perimeter provided the most important and informative input in the phenotype classification based on cellular morphology. For classification based on the colonial data, we found that colonial Area and Area of intercellular space were the most informative. When the cellular and colonial parameters were combined, colonial Feret’s diameter, colonial Minimal Feret’s diameter, and colonial Shape factor had the greatest impact on classification.

This information can be used in two ways. Firstly, new biological knowledge can be obtained by focusing on the molecular mechanisms associated with the change in the important features under spontaneous differentiation. Secondly, simplified classification models can be trained to confine the predictors to only the important ones. This can be especially useful when a much larger amount of data are involved, so that the computational efficiency becomes a bottleneck.

The analysis of feature importance on the combined cellular and colonial data suggests that the morphological properties of colonies play a major role in assessing the phenotype. The shallow distribution of the importance score for cellular parameters in the best model based on the combined data indicates that each cellular morphological feature adds some information to the whole picture, but no single parameter can be singled out as drastically more informative.

The high classification accuracy of 98–99% that we have obtained approaches and sometimes exceeds the performance scores of previously reported classification models applied to pluripotent stem cells [11,12,15,16,17,18,19,20,21,22,23,24]. Morphological parameters of cells and colonies used as predictors in our models are biologically interpretable but require methods for their extraction from the images prior to classification. Other morphological characteristics, including morphological features of intracellular objects, have previously been considered and resulted in a classification accuracy of 80–89% [16,18]. Methods for automated feature extraction from images and videos of hPSCs with the subsequent application of supervised ML algorithms constitute another approach, with the reported classification accuracy values higher than 87% [19,20,21,24]. DL-based classification models applied directly to the images of hPSCs have been reported to perform at about 90% accuracy [12,23].

Despite the good performance shown by the classification models on the combined data, our approach has several limitations. We used data from three cell lines, and this number should be increased to make the models more applicable. This requires further studies on collecting morphological and phenotypic information for various hPSC lines, since previous efforts in developing classification models involved similar numbers of cell lines [13]. To make classification even more general, multiple hPSC growing conditions, including various experimental matrices and media, should also be tested. Another limitation concerns the necessity of extracting the morphological features prior to the application of our classification models, as this extraction is not a part of the models reported here. As a possible alternative, DL-based image classification can be utilized, in which no prior feature extraction is usually required [12,23].

Overall, our study confirms the utility of ML methods for the automated phenotype prediction for various hPSC lines. We consider our research as the first step towards developing software-guided analytical tools (Figure 1) that will automate the selection of the best iPSC clone for further research, namely for targeted differentiation of a patient-specific iPSC line towards the desired tissue-specific cell type. One of the bottlenecks in the use of iPSCs is the fact that not all obtained patient-specific clones are able to differentiate in the desired tissue-specific direction with equal efficiency. We previously showed the relationship between the morphological parameters of clones with different morphological phenotypes and the ability to differentiate along three germ layers [11]. In this study, we further refined our models to improve the efficiency of selecting the best clone. Based on these data, we are currently testing our model on clones that are unable to differentiate efficiently into mesenchymal stem cells and cardiomyocytes to improve model sensitivity.

## Figures and Tables

**Table 1:** Classification model performance for cellular and colonial data and for various classification methods. Nested cross-validation accuracy and area under the ROC-curve (ROC AUC) are shown as measures of performance. Best performance values are highlighted in bold.

Method	Cellular Data		Colonial Data	
	Accuracy	ROC AUC	Accuracy	ROC AUC
Naïve Bayes	58 ± 2%	0.69	60 ± 14%	0.71
*k*-nearest neighbors	64 ± 3%	0.66	68 ± 12%	0.71
Logistic regression	59 ± 4%	0.63	**75 ± 12%**	**0.90**
Random forest	64 ± 2%	0.67	66 ± 10%	0.79
Support vector machines	64 ± 3%	0.68	68 ± 11%	0.86
Artificial neural networks	**67 ± 4%**	**0.70**	71 ± 12%	0.89

**Table 2:** Nested cross-validation accuracy in classification models trained on cell-line-specific data.

	Accuracy		
	hESC H9	hiPSC AD3	hiPSC CaSR
Cellular data (artificial neural networks)	73 ± 7%	60 ± 6%	64 ± 3%
Colonial data (logistic regression)	75 ± 18%	62 ± 20%	67 ± 17%

**Table 3:** Classification model performance for combined cellular and colonial data and for various classification methods. Nested cross-validation accuracy and area under the ROC-curve (ROC AUC) are shown as measures of performance. Best performance values are highlighted in bold.

Method	Accuracy	ROC AUC
Naïve Bayes	72 ± 7%	0.815
*k*-nearest neighbors	88 ± 4%	0.818
Logistic regression	80 ± 6%	0.826
Random forest	**99 ± 2%**	**0.997**
Support vector machines	96 ± 2%	0.975
Artificial neural networks	**98 ± 2%**	0.956

## Data Availability

Data with values of morphological parameters of cells and colonies extracted from phase-contrast images of three cell lines (H9, AD3, and HPCASRi002-A) were downloaded from the Zenodo public repository (https://doi.org/10.5281/zenodo.7150644, accessed on 1 February 2023) [25]. Programs implementing the classification models developed in this study were uploaded to the Zenodo public repository (https://zenodo.org/records/10052095, accessed on 30 October 2023).
